# Livin expression is an independent factor in rectal cancer patients with or without preoperative radiotherapy

**DOI:** 10.1186/1748-717X-8-281

**Published:** 2013-12-02

**Authors:** Zhen-Yu Ding, Hong Zhang, Gunnar Adell, Birgit Olsson, Xiao-Feng Sun

**Affiliations:** 1 Division of Oncology, Department of Clinical and Experimental Medicine, Faculty of Health Sciences, Country Council of Östergötland, University of Linköping, Linköping, Sweden; 2 Department of Thoracic Oncology, Cancer Center, and the State Key Laboratory of Biotherapy, West China Hospital, West China Medical School, Sichuan University, Chengdu, China; 3 School of Medicine, Örebro University, SE 701 82 Örebro, Sweden; 4 Department of Oncology, Karolinska University Hospital, Stockholm, Sweden

**Keywords:** Rectal cancer, Livin, Radiotherapy

## Abstract

**Background:**

This study was aimed to investigate the expression significance of Livin in relation to radiotherapy (RT), clinicopathological and biological factors of rectal cancer patients.

**Methods:**

This study included 144 primary rectal cancer patients who participated in a Swedish clinical trial of preoperative radiotherapy. Tissue microarray samples from the excised primary rectal cancers, normal mucosa and lymph node metastases were immunostained with Livin antibody. The proliferation of colon cancer cell lines SW620 and RKO was assayed after Livin knock-down.

**Results:**

The expression of Livin was significantly increased from adjacent (*P* = 0.051) or distant (*P* = 0.028) normal mucosa to primary tumors. 15.4% (2/13) and 39.7% (52/131) patients with Livin-negative and positive tumors died at 180 months after surgery, and the difference tended to be statistically significant (*P* = 0.091). In multivariate analyses, the difference achieved statistical significance, independent of TNM stage, local and distant recurrence, grade of differentiation, gender, and age (odds ratio = 5.09, 95% CI: 1.01-25.64, *P* = 0.048). The in vitro study indicated colon cancer cells with Livin knock-down exhibited decreased proliferation compared with controls after RT.

**Conclusions:**

The expression of Livin was was independently related to survival in rectal cancer patients, suggesting Livin as a useful prognostic factor for rectal cancer patients.

## Background

Colorectal cancer (CRC) is one of the leading causes of cancer death in Western countries [1,2]. Surgery remains the curative modality for the CRC, and preoperative radiotherapy (RT) has shown a survival advantage compared with surgery alone. However, the value of preoperative RT still remains controversial [3-5]. There is an urgent need to search for predictive indicators to identify patients who can be benefited from preoperative radiotherapy.

Many molecules that participate the biological process of proliferation and apoptosis have been proposed as potential indicators for RT. We have previously reported that Survivin from the Inhibitor of Apoptosis (IAP) family is an independent prognostic factor in rectal cancer patients with or without preoperative radiotherapy [6]. Till now, another IAP family member Livin has been identified [7-9]. It is expressed in variant tumors such as melanoma, leukemia, bladder cancer, breast cancer, cervical cancer, nasopharyngeal cancer and lung cancer [10-13]. Two isoforms (designated α- and β-) were described due to alternative splicing, which are almost identical except for a 54 bp truncation in exon 6 [14]. Both isoforms block apoptosis induced by TNF-α and anti-CD95 antibody.

However, little is known on the Livin expression in CRC except for preliminary in vitro reports [15]. Neither is it known about the relationship between Livin expression and radiotherapy. In this study, we investigated the relationships of Livin expression to radiotherapy and to clinicopathologic or biologic variables in CRC patients who participated in a clinical trial of preoperative radiotherapy.

## Patients and methods

### Patients

The current study got approval from the ethics committee of Linkoping University and was in compliance with the Helsinki Declaration. The present study included 144 rectal cancer patients from the Southeast Swedish Health Care region who participated in the Swedish Rectal Cancer Trial between 1987 and 1990 [3]. Of the 144 patients, 77 underwent tumor resection alone and 67 underwent preoperative RT and tumor resection. None of the patients had received chemotherapy before surgery. Besides the tumor specimens, matched normal mucosa adjacent to the tumor tissue were collected from 71 cases, distant (4-35 cm from the primary tumor) normal mucosa were collected from 110 cases, and metastases in the regional lymph nodes were collected from 47 cases. The mean patient age was 66 years (range, 36-85 years). The median follow-up was 85 months. RT was given to 25 Gy in five fractions during a median of 8.5 Days (range, 6-18 days) [3]. Surgery was then performed in a median of 3.4 days (0-11 days) after RT. The mean tumor distance to the anal verge was 7.4 cm in the surgical group and 8.6 cm in the surgery plus RT group (*P* = 0.10). Other patient and tumor characteristics were presented in Table 1. No statistically significant differences were found between the two groups.

**Table 1:** Characteristics of patients and tumors

**Characteristics**	**Non radiotherapy n (%)**	**Radiotherapy n (%)**
Gender		
Male	44 (57)	42 (63)
Female	33 (43)	25 (37)
Age (years)		
≤70	45 (58)	45 (67)
>70	32 (42)	22 (33)
TNM stage		
I	19 (25)	20 (30)
II	19 (25)	22 (33)
III	35 (45)	19 (28)
IV	4 (5)	6 (9)
Differentiation		
Good	60 (78)	49 (73)
Poor	17 (22)	18 (27)
Number of malignancies		
Single	65 (84)	55 (82)
Multiple	10 (13)	12 (18)
Unknown	2 (3)	0
Surgical type		
Rectal amputation	42 (55)	24 (36)
Anterior resection	35 (45)	43 (64)
Resection margin		
Tumor free	73 (95)	63 (94)
Tumor	4 (5)	4 (6)
Distance to anal verge (cm)		
Mean	7.4	8.6

### Immunohistochemical (IHC) assay

Representative paraffin-embedded tissue blocks were selected for the tissue microarray. Three morphologically representative regions were chosen in each block and three cyclindrical core core tissue specimens (0.6 mm in diameter) were taken from these areas, inserted in another paraffin block. Sections from the second block were cut into 5 μm chips using a microtome, mounted on microscopic slides. The tissue microarrays were constructed using a manual arrayer (Beecher Inc., WI).

IHC for Livin expression was done on 5-μM tissue microarray sections from paraffin-embedded surgical specimens. The sections were baked in an oven at 60°C for over 6 hrs and then deparaffinized with xylene and rehydrated with a series of decreasing concentrations of ethanol. To demask antigen epitopes, the sections were soaked in DIVA solution (Biocare Medical, CA) in a high pressure cooker at 125°C for 30 sec after which the sections were cooled to 90°C for 10 sec and then kept in room temperature for 30 min followed by washing in phosphate buffered solution (PBS, pH 7.4). To inhibit endogenous peroxidase activity, the sections were incubated with 3% H2O2-methanol for 20 min. After blocking with power block solution (Spring Bioscience, CA) for 10 min, the sections were incubated with goat anti-Livin antibody (RnD, MN) at a concentration of 2.5 μg/ml at 4°C overnight. EnVision anti-goat Polymeric conjugate (Dako, Carpinteria, CA) was subsequently applied for 30 min. The slides were washed in PBS and the peroxidase reaction was performed using 3,3’-diaminobenzidin (Sigma Chemical, St. Louis, MO) and 3% H2O2. Finally, hematoxylin was used for counterstaining. Sections known to show positive staining for Livin were included for each turn, receiving either the primary antibody or control isotype Ig, as positive or negative controls, whereas there was no staining in the negative controls.

### Measurement of the Livin expression by IHC

The IHC results of the Livin in tissue specimens were the mean of scores by two independent authors (Z.-Y. D., and H. Z., who is a pathologist) in a blinded fashion without knowledge of the clinicopathological or biological information. Each investigator estimated the proportion of cells stained and the intensity of staining in the whole section. The intensity in epithelial cells or tumor cells was scored as 0 (negative staining), 1 (weak staining exhibited as light yellow), 2 (moderate staining exhibited as yellow brown), and 3 (strong staining exhibited as brown). If there was a discrepancy in individual scores, then both investigators re-evaluated the slides together to reach a consensus before combining the individual scores. To avoid an artificial effect, the cells on the margins of the sections and in areas with poor morphology were not counted.

### Evaluation of proliferation, P53, mammary tumor 8 kDa (MAT8), ataxia telangiectasia mutated (ATM), apoptosis, and necrosis

Proliferation in the cancer cells was measured using IHC for Ki-67 as an indicator (n = 115). Low and high proliferation were defined in sections where <32% or ≥32% of cancer cells expressed Ki-67 [16]. The data for P53 (n = 139), MAT8 (n = 124) and ATM (n = 66) of primary rectal cancers determined by IHC were taken from previous studies performed with the same cases used in the present study at out laboratory [17-19]. Apoptosis was detected by the terminal deoxynucleotidy transferase-mediated dUTP-biotin nick end labeling (TUNEL) assay [20].

### Cell culture

Human colon cancer cell lines RKO and SW620 were purchased from the American Type Culture Collection. Cells were maintained in Minimal Essential Medium (Sigma-Aldrich) supplemented with 10% FBS (GIBCO, Invitrogen, Carlsbad, USA), 1.5 mM L-glutamine (GIBCO) and 1X PEST (GIBCO) at 37°C in a 5% CO2 incubator. Mycoplasma contamination was excluded from the cells by using a commercially available PCR kit (PromoKine, Heidelberg, Germany). The endogenous Livin was knock-down with ON-TARGETplus^R^ SMARTpool siRNA against Livin (L-004391-00-5551, Thermo Fisher) by using DharmaFect 2 transfection reagent (Dharmacon) according to the manufacturer’ instructions. The transfection mixture consisted of antibiotic-free culture medium and 100 nM siRNA. The Dharmacon Non-Targeting scramble siRNA was used as a negative control.

### Cell proliferation assay

The effect of radiotherapy on the proliferation of colon cancer cells was quantified by WST-1 assay (Roche) according to the manufacture’s instructions. Briefly, cells were seeded in 96-well tissue culture plate, in 100 μl culture medium. 10 μl of WST-1 assay solution was added to each well and the cells were further incubated at 37°C for 2-3 hrs. The absorbance was measured at a wavelength of 450 nm on a VersaMax microplate reader (Molecular Devices, Sunnyvale, CA). Untreated cells served as the indicator of 100% cell viability.

### Statistical analysis

All the statistical analyses were performed by using the SPSS software 19.0 (IBM software). The McNemar and Chi-Square methods were used to test the statistical significance of the differences in the Livin expression between different tissue specimens and the association of Livin with the clinicopathological features, Ki-67, P53, MAT8 and ATM. Cox’s proportional hazard model used to estimate the relationship between Livin expression and survival, including both univariate and multivariate analyses. Survival curves were computed according to the Kaplan-Meier method. Tests were two-sided, and *P* < 0.05 was considered statistically significant.

## Results

### Livin expression in normal mucosa, primary tumor and lymph node metastasis

**Figure 1:**
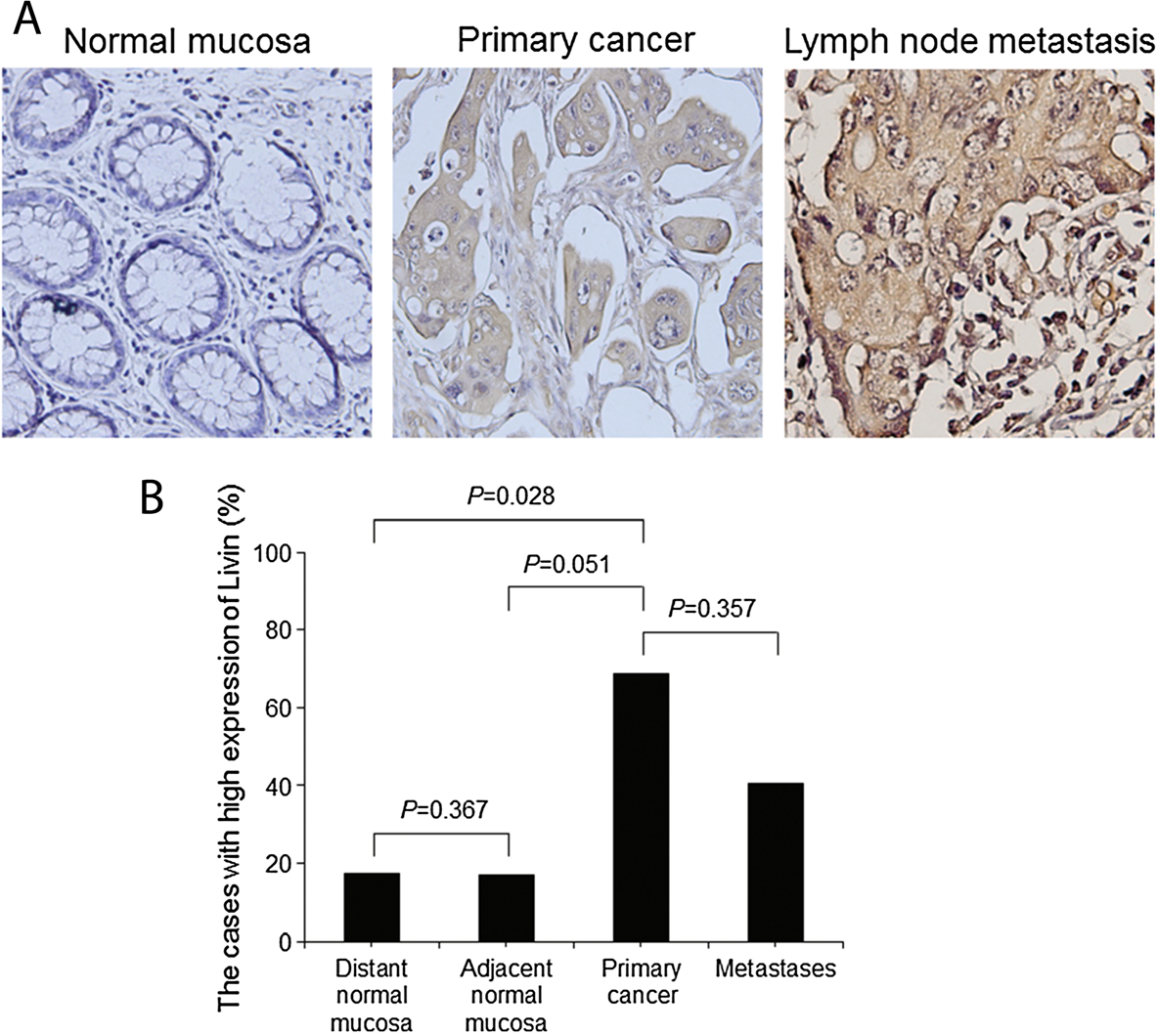
**Livin expression in tumors and mucosa.** Livin expression in normal mucosa, primary cancer, and lymph node metastases **(A)**. The percentage of Livin high expresssion differes significantly between primary cancer and adjacent or distant normal mucosa **(B)**.

By immunostaining, Livin expression was predominantly detected in the cytoplasm of epithelial cells of normal mucosa, and tumor cells of primary cancers and lymph node metastases, with little staining in the nuclei (Figure 1A and Additional file 1). For further analysis of the study, only the staining of the cytoplasmic Livin was measured and presented. Of the 144 primary tumors examined, Livin expression was negative in 13 cases (9%), weak in 32 cases (22%), moderate in 72 cases (50%), and strong in 27 cases (19%). The frequency of high expression (moderate or strong staining) of Livin was significantly increased from distant normal mucosa (17%, 19/110; *P* = 0.028) or adjacent normal mucosa (17%, 12/71; *P* = 0.051) to primary tumors (69%, 99/144), while the expression was not different from primary tumors to lymph node metastases (40%, 19/47; *P* = 0.357). There was no significant difference between distant and adjacent normal mucosa (*P* = 0.367, Figure 1B).

### Effect of preoperative RT on Livin expression in the normal mucosa, primary cancer and lymph node metastasis

Compared with the cases without preoperative RT, the frequency of Livin-positive expression in primary cancers with preoperative RT was decreased from 97% to 83% (*P* = 0.004), while in the distant normal mucosa, the frequency of Livin-positive expression was increased (71% VS 89%, *P* = 0.021). However, Livin-positive cases in adjacent normal mucosa were not significantly influenced by RT (75% VS 76%, *P* = 0.931). The frequency of Livin-positive cases in the lymph node metastases was unchanged in cases receiving preoperative RT (86% VS 90%, *P* =0.986).

### Livin expression in relation to clinicopathological and biological factors

**Figure 2:**
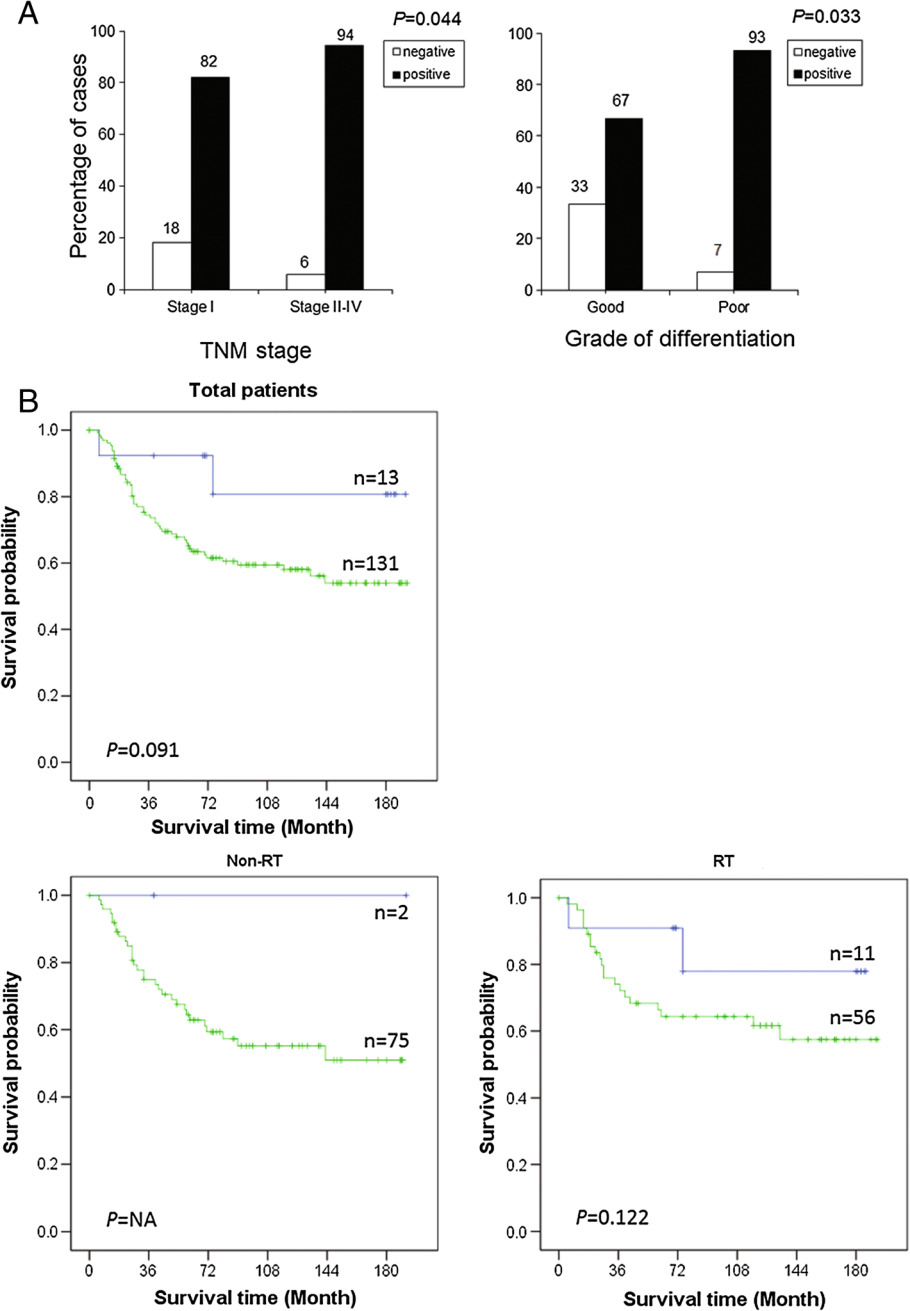
**Livin expression in relation to clinicopathological factors.** Significant difference in Livin expression between early to advanced stage or good to poor differentiation **(A)**. Livin expression in relation to survival in entire group and subgroup with or without radiotherapy **(B)**.

In primary cancers, the expression of Livin was associated with lower frequency of stage I and higher frequency of stage II, III or IV cases (*P* = 0.044), and related to poor differentiation of cancers (*P* = 0.033, Figure 2A). Patients with Livin-positive or negative tumors had similar local (*P* = 0.647) and distant (*P* = 0.280) recurrence. The expression of Livin was not related to gender, or age (*P* > 0.05). 15.4% (2/13) and 39.7% (52/131) patients with Livin-negative or positive tumors died at 180 months after surgery, and the difference tended to be statistically significant (*P* = 0.091, Figure 2B). In multivariate analyses, the difference achieved statistical significance, independent of TNM stage, local and distant recurrence, grade of differentiation, gender, and age (*P* =0.048, Table 2).

**Table 2:** multivariate analysis of Livin in tumors in relation to survival

**Factor**	**Patients (n)**	**Cancer death rate ratio (95% confidence interval )**	***p *** **value**
Livin expression			0.048
Negative	13	1.0	
Positive	131	5.09 (1.01-25.64)	
Gender			0.917
Male	86	1.0	
Female	58	0.97 (0.52-1.79)	
Age (years)			0.779
≤70	90	1.0	
>70	54	1.08 (0.62-1.90)	
Tumor stage			<0.001
I + II + III	134	1.0	
IV	10	5.40 (2.13-13.69)	
Differentiation			0.881
Poor	138	1.0	
Good	6	1.10 (0.31-3.99)	
Local recurrence			<0.001
No	121	1.0	
Yes	23	3.31 (1.78-6.17)	
Distant recurrence			<0.001
No	85	1.0	
Yes	59	12.48 (5.60-27.75)	

In a subgroup analysis of the patients with preoperative RT, a statistically significant difference was observed between patients with Livin-positive or negative tumors (*P* = 0.047). We did not find any relationship of Livin expression to TNM stage, grade of differentiation, gender, or age in this subgroup (*P* >0.05). Due to a few cases with Livin-negative tumors in the subgroup of the patients without preoperative RT, we did not perform statistical analysis in the subgroup.

In the entire group, the frequency of expression of Livin was related to the expression of Ki-67 (*P* =0.027), p53 (*P* =0.012), MAT8 (*P* =0.020) and inversely related to ATM (*P* =0.007).

**Figure 3:**
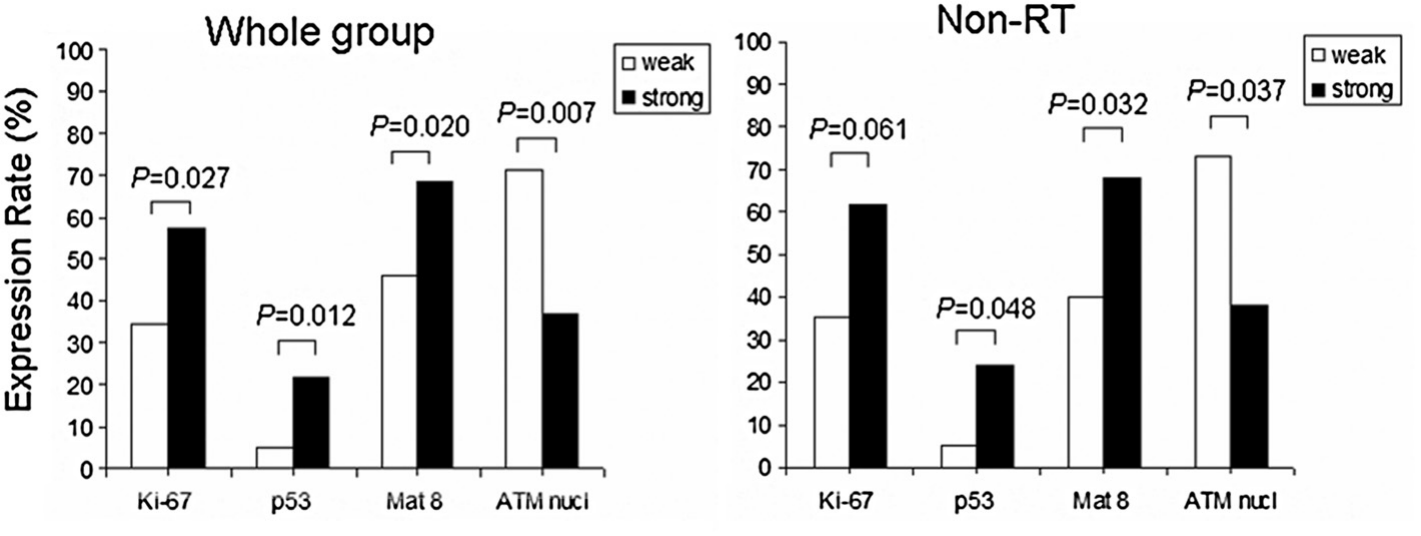
**Livin expression in relation to other factors.** The expression of Livin was related to the expression of Ki-67, p53, MAT8 and inversely related to ATM in both the entire group and non-RT subgroup patients.

In the patients without preoperative RT, high expression of Livin was still related to MAT8 (*P* =0.032) and p53 (*P* =0.048), and inversely related to ATM (*P* =0.037, Figure 3). In the patients with preoperative RT, no trend was observed toward any of the relationship (*P* >0.05).

In the current study, the median overall survival was similar between patients with (129 months) or without (114 months) preoperative RT (*P* = 0.252).

### Knock-down of Livin inhibited the proliferation of colon cancer cells after irradiation

**Figure 4:**
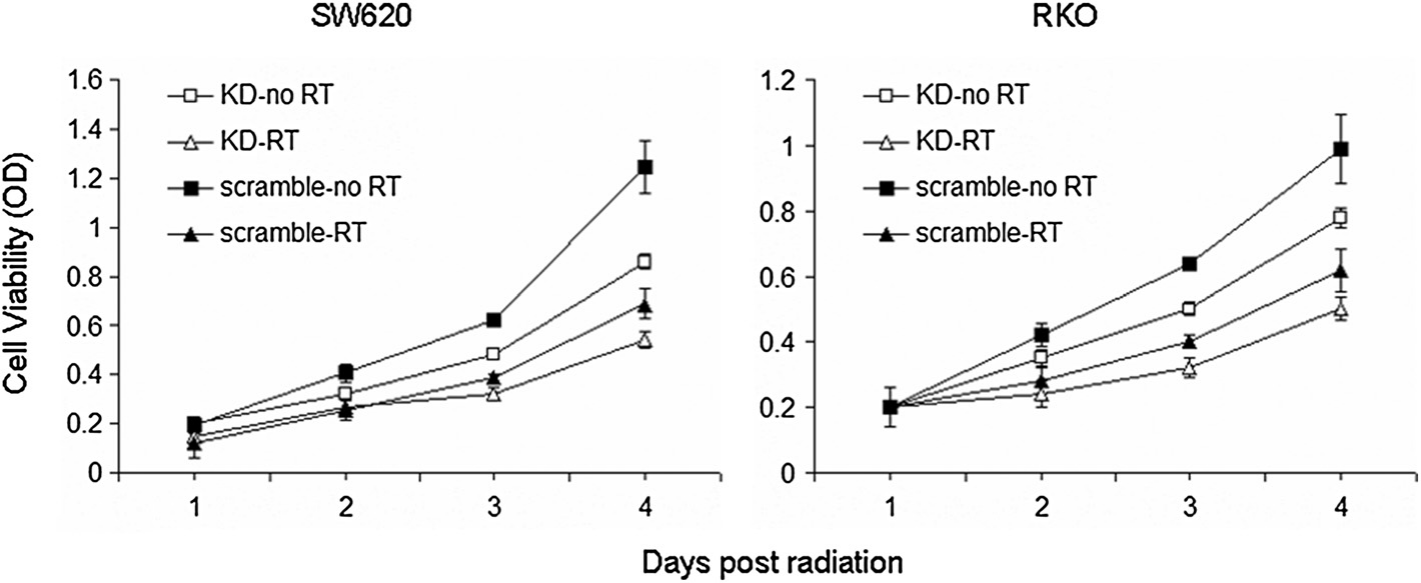
Knock-down of Livin makes cells more vulnerable to RT.

The endogenous Livin in SW620 and RKO colon cancer cells was knocked-down by siRNA. The non-targeting scramble siRNA was used as controls. The proliferation of cancer cells after irradiation was monitored continuously by WST-1 method. The colon cancer cells with Livin knock-down exhibited significant decrease in proliferation at each time point, compared scramble siRNA treated controls (*P* <0.05, Figure 4).

## Discussion

This is the first study of Livin expression in rectal cancer patients who participated in a trial of preoperative RT. Livin expression was described in colon cancer cell lines at the protein or mRNA level [15,21-23], however few reported the expression of Livin in rectal cancer tissues. Yagihashi et al reported the detection of anti-Livin autoantibody in gastrointestinal patients, but this study failed to confirm the Livin expression in tumor tissues either [24]. In the present study, we found the extensive expression (91%) of Livin in CRC tissue specimens. We also detected Livin expression in 7 from 7 colon cancer cell lines [25]. The prevalent expression of Livin in CRC tissues and cells suggested it plays a prominent role for the progression of CRC.

Controversies remained on the prognostic value of Livin in different tumor types. Livin over-expression together with C-myc amplification in patients with neuroblastoma predicated a poor prognosis [26]. In patients with bladder cancer, increased Livin expression in tumors was associated with shorter duration of relapse-free survival [11]. Livin expression was also parallel with a decreased overall survival in osteosarcoma patients [27]. Although most studies supported the negative impact of Livin on survival, mild influence or even favored prognosis was also reported. One paper implicated the Livin expression was a favorable prognostic factor in childhood acute lymphoblastic leukemia [12]. However, the authors did not provide explanations for the counter-instinctive results. In summary, it would be reasonable to suggest the prognostic significance of Livin is tissue and tumor specific. In the current study, we found that Livin expression was an independent prognostic factor for CRC patients after adjustment for TNM stage, local and distant recurrence, grade of differentiation, gender, and age. Our results provide new evidence in support of the important roles played by Livin in variant cancers.

In the present study, we individually examined the relationship between Livin expression and survival in the patients with or without preoperative RT. Our results showed Livin expression in both the entire group and the subgroup of patients receiving preoperative RT was statistically related to poor prognosis. In the subgroup of patients without preoperative RT, the expression of Livin seemed to be related to the shorter overall survival. The survival rate in patients with Livin-negative cancers was 100% (2/2) at 200 months after surgery. These evidence supported the notion that Livin was related to survival in CRC patients regardless of receiving preoperative RT or surgery alone.

Few studies reported the differential expression of Livin between primary cancers and adjacent normal mucosa. In the current study, we found Livin was over-expressed in 69% of primay cancers and 40% of lymph node metastases, but only 17% of adjacent normal mucosa and 17% of distant normal mucosa. The frequency of high expression of Livin was much higher in malignant tissues than in normal mucosa. Studies performed in cancer cells lines implicated the expression of Livin was under the regulation of Catenin/TCF or mTOR pathways [28,29] which were considered hallmark signaling changes in cancers [30]. These studies together with our results indicated the Livin deregulation may contribute to the malignant manifestation in cancers and may serve as a potential therapeutic targets [31].

We analyzed the influence of preoperative RT on the Livin expression, and observed a decrease of Livin expression in primary cancers in patients receiving preoperative RT. Cancers have deregulated apoptotic pathway to protect them against harmful stimuli including radiation [30]. Anti-cancer treatment would overcome these molecular hurdles to be effective [32]. In this way we believe Livin was down-regulated by the preoperative RT in primary cancers. The adjacent normal mucosa was suggested a place of intermediate biologic change and had a similar tendency with the primary cancers. However, Livin in the distant mucosa might be up-regulated to protect the cells from apoptosis by RT.

The underlying mechanism of Livin expression in association with poor prognosis was probably attributed to the negative regulation of apoptosis in response to RT. Livin was proposed to block apoptosis by inhibiting the activity of caspases [7,8] or involved in the TAK1/JNK1 pathway [33]. In the present study, we did not prove the relationship between Livin expression and apoptosis in the rectal cancer tissues. Similarly, our previous study on Survivin did not prove the relationship to the apoptosis rate either [6]. One of the reasons may be due to the “surgery-related apoptosis” which might influence our results concerning apoptosis in relation to Livin expression. We further analyzed the relationship of Livin with Ki-67, P53, MAT8, and ATM. Ki-67 is a proven indicator of cell proliferation [16]. P53 detected in IHC is a mutated protein with oncogene properties [17]. MAT8 also named FXYD-3, is a chloride channel or chloride channel regulator and acts as a prognostic factor for cancers [18]. Livin expression was inversely related to ATM which is a serine/threonine protein kinase in the DNA damage repair pathway [34]. Our findings suggested Livin might be involved in variant pathways in the cell malignant phenotypes.

It should be noted that debate remained as to whether apoptosis contributed to radiotherapy [35]. It was proposed that apoptosis contributed to the short-term cytocidal effects of radiotherapy, but not to the long-term clone formation. In our study, we found the expression of Livin was related to the prognosis in patients. We wouldn’t be able to find the association between Livin expression and apoptosis in this cohort of patients. These results were in good agreement to the proposal mentioned above. Therefore, the mechanistic explanation for the current observations couldn’t be simply attributed to apoptosis. Livin might be involved in other phenotypes too. In support of this, a paper described its regulation in cell cycle was published [36].

Our in vitro study showed decreased proliferation of SW620 and RKO colon cancer cells after radiation when the endogenous Livin was knocked down by RNAi. Consistent with our findings, recent studies provided evidence to support the role of Livin for treatment resistance. Crnkovic-Mertens et al reported silencing Livin expression strongly increased the apoptotic rate in response to different stimuli [37,38]. Besides, Wang et al showed silencing Livin inhibited the proliferation of tumor cells [22]. The inhibition of proliferation was probably related to cell cycle regulation [36]. Our previous results also showed knock-down of Livin rendered the colon cancer cells more sensitive to chemotherapy agent cisplatin [25]. The inhibiting role of Livin in colon cancer cells was consistent with the immunostaining results, where Livin was found to be related to poor prognosis.

Of notice, the patients in our study were from a previous randomized trial [3]. Although they were well-balanced between groups (Table 1), the sample size was restricted to the available banked tissue samples. The limited number of patients prevented any conclusive results. Additionally, although our multivariate analysis might be helpful, the problem of the heterogeneity in patients (stage I-IV) could not be completely solved. All these limitations indicated the results should be interpreted cautiously.

In summary, our study showed increased Livin expression in primary rectal cancers was related to the more advanced stage of cancer. Livin expression was independently related to survival in rectal cancer patients who participated in a trial of preoperative RT, and it was associated with survival in subgroup of patients receiving preoperative RT too. Livin expression tended to be down regulated by RT. Taken together, our data implicated Livin was a useful prognostic factor for rectal cancer patients and possibly served as a potential therapeutic target.

## Consent

Written informed consent was obtained from the patient for the publication of this report and any accompanying images.

## Abbreviations

RT: Radiotherapy; CRC: Colorectal cancer; IAP: Inhibitor of apoptosis.

## Competing interests

The authors declare that they have no competing interests.

## Authors’ contributions

DZ collected and analyzed the data and prepared the manuscript; ZH participated in the analyzing the IHC data; AG and OB provided the critical revision of the manuscript and the administrative support; SX provided the conception of the study and the final approval of the final version. All authors read and approved the final manuscript.

## Supplementary Material

Additional file 1 **IHC staining of Livin.** Typical presentation of weak (A) and strong (B) staining signals of Livin. Click here for file
