# Supplementary figures and images for: Livin expression is an independent factor in rectal cancer patients with or without preoperative radiotherapy
Source: Radiat Oncol. 2013 Dec 2;8:281. doi: 10.1186/1748-717X-8-281 (PMC3904757; doi:10.1186/1748-717X-8-281)

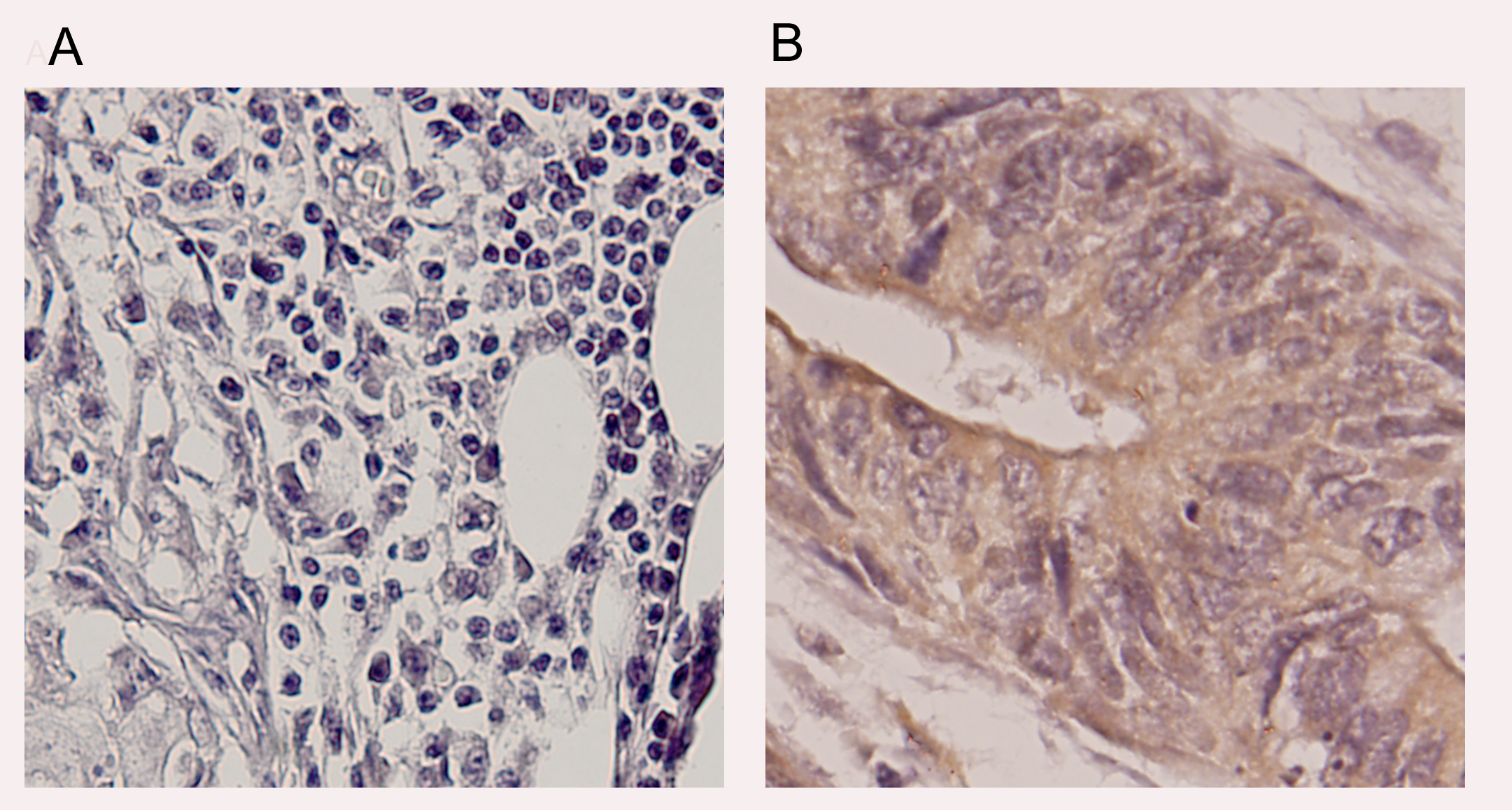

Supplement: Additional file 1 — IHC staining of Livin. Typical presentation of weak (A) and strong (B) staining signals of Livin. [file 1748-717X-8-281-S1.tiff]
